# Immune-instructive copolymer scaffolds using plant-derived nanoparticles to promote bone regeneration

**DOI:** 10.1186/s41232-022-00196-9

**Published:** 2022-04-03

**Authors:** Salwa Suliman, Anna Mieszkowska, Justyna Folkert, Neha Rana, Samih Mohamed-Ahmed, Tiziana Fuoco, Anna Finne-Wistrand, Kai Dirscherl, Bodil Jørgensen, Kamal Mustafa, Katarzyna Gurzawska-Comis

**Affiliations:** 1 Centre of Translational Oral Research (TOR), Department of Clinical Dentistry, Faculty of Medicine, University of Bergen, Årstadveien 19, 5009 Bergen, Norway; 2 Department of Microbiology, Faculty of Biochemistry, Biophysics and Biotechnology, Jagiellonian University, 31-007 Krakow, Poland; 3 Department of Fibre and Polymer Technology, School of Engineering Sciences in Chemistry, Biotechnology and Health, KTH Royal Institute of Technology, Stockholm, Sweden; 4 Dansk Fundamental Metrologi A/S, Kogle Allé 5, 2970 Hørsholm, Denmark; 5 Department of Plant and Environment Sciences, Section for Plant Glyco Biology, University of Copenhagen, Copenhagen, Denmark; 6 Department of Oral Surgery, Institute of Clinical Sciences, College of Medical & Dental Science, The University of Birmingham, 5 Mill Pool Way, Birmingham, B5 7EG UK; 7 Birmingham Community Healthcare NHS Foundation Trust, Birmingham Dental Hospital, Oral Surgery Department, Birmingham, UK

**Keywords:** Plant-derived RG-I, Copolymer, Pectin, Immunomodulation, Inflammation

## Abstract

**Background:**

Age-driven immune signals cause a state of chronic low-grade inflammation and in consequence affect bone healing and cause challenges for clinicians when repairing critical-sized bone defects in elderly patients.

**Methods:**

Poly(l-lactide-co-ɛ-caprolactone) (PLCA) scaffolds are functionalized with plant-derived nanoparticles from potato, rhamnogalacturonan-I (RG-I), to investigate their ability to modulate inflammation in vitro in neutrophils and macrophages at gene and protein levels. The scaffolds’ early and late host response at gene, protein and histological levels is tested in vivo in a subcutaneous rat model and their potential to promote bone regeneration in an aged rodent was tested in a critical-sized calvaria bone defect. Significant differences were tested using one-way ANOVA, followed by a multiple-comparison Tukey’s test with a *p* value ≤ 0.05 considered significant.

**Results:**

Gene expressions revealed PLCA scaffold functionalized with plant-derived RG-I with a relatively higher amount of galactose than arabinose (potato dearabinated (PA)) to reduce the inflammatory state stimulated by bacterial LPS in neutrophils and macrophages in vitro*.* LPS-stimulated neutrophils show a significantly decreased intracellular accumulation of galectin-3 in the presence of PA functionalization compared to Control (unmodified PLCA scaffolds). The in vivo gene and protein expressions revealed comparable results to in vitro. The host response is modulated towards anti-inflammatory/ healing at early and late time points at gene and protein levels. A reduced foreign body reaction and fibrous capsule formation is observed when PLCA scaffolds functionalized with PA were implanted in vivo subcutaneously*.* PLCA scaffolds functionalized with PA modulated the cytokine and chemokine expressions in vivo during early and late inflammatory phases. PLCA scaffolds functionalized with PA implanted in calvaria defects of aged rats downregulating pro-inflammatory gene markers while promoting osteogenic markers after 2 weeks in vivo.

**Conclusion:**

We have shown that PLCA scaffolds functionalized with plant-derived RG-I with a relatively higher amount of galactose play a role in the modulation of inflammatory responses both in vitro and in vivo subcutaneously and promote the initiation of bone formation in a critical-sized bone defect of an aged rodent. Our study addresses the increasing demand in bone tissue engineering for immunomodulatory 3D scaffolds that promote osteogenesis and modulate immune responses.

**Supplementary Information:**

The online version contains supplementary material available at 10.1186/s41232-022-00196-9.

## Background

Global average age expectancy is increasing [1]. This leads to an inevitable increase in chronic age-related diseases [2], such as osteoporosis and osteoarthritis [3].

Age-driven immune signals cause a state of chronic low-grade inflammation and in consequence affects the potency of endogenous stem cells [4]. This could explain the challenges facing surgeons while reconstructing critical-sized bone defects in elderly patients. These very often require bone augmentation using an autogenous bone graft, which is associated with clinical drawbacks, related to limited tissue availability, that increase patient morbidity [5] and risk of infections at the donor surgical site [6]. Thus, there is an increasing demand in bone tissue engineering for immunomodulatory three-dimensional (3D) scaffolds that promote osteogenesis and modulate immune responses, by delivering bioactive factors. The success of bone healing is vastly determined by the initial inflammatory phase, which is affected by both the local and systemic factors. Moreover, bone healing is modulated by intracellular pathways and cell-to-cell communications [7]. Cells that play the main role in modulating bone healing are immune cells and progenitor cells [8]. Any discrepancy in the number or activity of these cells may cause a prolonged inflammatory response leading to chronic inflammation that can impair the new bone formation.

Recent studies demonstrated the immunomodulatory properties of plant-derived molecules, mainly represented by the polysaccharide, called rhamnogalacturonan-I (RG-I) [9]. The RG-I is a subunit of pectin composed of a backbone of alternating rhamnose and galacturonic acid residues, with arabinose and galactose side chains present on the rhamnosyl residues. The RG-I structure mimics the polysaccharides from the extracellular matrix of mammals [10] and therefore has been proposed as a bioactive molecule to stimulate cell response during bone healing [11]. Recent studies showed that specifically RG-I with a relatively high content of galactose (Gal) compared to the content of arabinose, stimulate adhesion, proliferation, and differentiation of macrophages [12], fibroblasts [13], osteoblasts [14–17], and bone marrow mesenchymal stromal cells [18, 19]. In addition, it has been reported that RG-I may also possess anti-inflammatory properties [12, 20–22]. The RG-I interaction with β-integrins prevents neutrophil adhesion to fibronectin, which represents a key step in the inflammatory response [20]. Also, the branched region of RG-I has been reported to be responsible for the proliferation of B lymphocytes [9]. Well-established methods for controlling the modification of pectin’s structure have opened new possibilities for using these plant-derived molecules as tissue engineering matrices [23, 24].

Copolymers, poly(l-lactide-co-ɛ-caprolactone) (PLCA), have been investigated as a promising material for bone tissue engineering by proving cytocompatibility and osteoconductivity both in vitro and in vivo [25]. They are inherently hydrophobic, and they lack native cell recognition sites [26], which makes their interaction with cells dependent on the unspecific adsorption of proteins from the surrounding biological fluids [25]. It is therefore desirable to functionalize PLCA intended for bone tissue engineering with specific bioactive signals. Several functionalizations, including modifications with nanodiamonds [27, 28], Tween 80 [29], or adsorption of bone morphogenetic protein 2 [30–32] or human demineralized dentine matrix [33] have been investigated to improve osteogenic properties. However, simultaneous targeting to the osteogenic and immune milieu has been a challenge. Therefore, modified RG-I to functionalize PLCA scaffolds would be a promising concept to stimulate immunomodulation and promote bone regeneration while lowering the risk of undesirable inflammation.

In this study, we functionalized PLCA scaffolds with RG-I to investigate their ability to modulate inflammation in vitro and in vivo, as well as to promote bone regeneration in an aged rodent model.

## Methods

### PLCA scaffold fabrication

The PLCA scaffolds were prepared using the solvent-casting particulate leaching method as previously described [31]. Copolymers (‘Resomer LC 703 S’, Evonik, Essen, Germany) were used with the number average molecular mass *M*_n_ = 142 kg mol^−1^ (*Ɖ* = 1.5) and composition in a mole ratio of 70 for l-lactide and 30 for ε-carprolactone. Scaffolds were punched out in different dimensions for in vitro and in vivo experiments. The scaffolds for in vitro studies were 12 mm in diameter and 1.3 mm in thickness and for in vivo scaffolds were 5 mm in diameter and 1.3 mm in thickness. The scaffold porosity was > 83% with an average pore size of 90–500 μm, measured by micro-computed tomography (Skyscan 1172, Bruker, MA, US) (40-kV and 2.4-μm voxel). Scaffolds were washed twice with ethanol 70%, followed by sterilization under ultraviolet light.

### Isolation of RG-I and functionalization of PLCA scaffolds with RG-I

RG-I was isolated and modified by an enzymatic treatment of the potato pulp as described previously [34]. Briefly, the arabinan side chains of unmodified potato RG-I (PU) were shortened with α-l-arabinofuranosidase and endo-arabinanase (Novozymes, Bagsværd, Denmark) and the modified form was named potato RG-I dearabinated (PA). The monosaccharide composition and linkage analysis of PU and PA have been reported previously [14, 34]. The PLCA scaffolds placed in multi-well polystyrene plates were physiosorbed with 500 μg/mL PU or PA and allowed to shake in a plate shaker (MixMate® Eppendorf, Germany) at 100 rpm overnight at room temperature. Unfunctionalized PLCA scaffolds were used as Control, while functionalized PLCA scaffolds with unmodified potato RG-I (PU scaffold) and with potato RG-I dearabinated (PA scaffold) were tested groups.

### Scaffold characterization

#### Atomic force microscopy

PLCA scaffolds (Control, PU, PA) were prepared for imaging with atomic force microscopy (AFM) by collecting small particles of varying sizes using a 15-blade scalpel. The particles were fixed to AFM discs of steel with an 18-mm diameter. The 3D scans were done using the AFM Force Microscope NX-20 (Park Systems, South Korea) with a feedback-controlled XY scan table and a maximum scan range of approximately 100 μm × 100 μm. It was equipped with a linearized Z-scanner with a maximum dynamic range of approximately 8 μm. A super-sharp scanning probe with a tip radius of nominally < 2 nm (SSS-NCH, Nanosensors, Switzerland) was applied to provide technical pixel resolution and to avoid feature blurring. The 3D scans were performed using the intermittent imaging technique. In addition to topographic features, variations of the interacting forces between the tip and the surface are recorded as changes in the phase signal of the tip oscillations in intermittent mode. The mapping of these force variations can help to localize different materials, for instance.

#### Confocal microscopy

PLCA scaffolds functionalized with PU and PA were visualized using immunofluorescence labelling and confocal microscopy. Functionalized and unfactionalized PLCA scaffolds were fixed with 4% paraformaldehyde for 10 min before blocking with 5% skimmed milk (pH 7.2) for 15 min. PLCA scaffolds were incubated with anti-(1→4)-β-galactan LM5 (Plant Probes, Leeds, UK) (1:10) at room temperature for 2 h with shaking. The antibody was diluted in 5% skimmed milk. Goat anti-rat fluorescein isothiocyanate (FITC) IgG (1:200) (Sigma-Aldrich, Munich, Germany) was used as a secondary antibody and incubated for 2 h with shaking. After washing with PBS, PLCA scaffolds were visualized using a Leica TCS-SP5 II confocal laser scanning microscope (Leica Microsystems, Exton, PA, USA) and images captured with PL FLUOTAR 10/× 0.30 dry objective.

### In vitro inflammatory evaluation

#### Cell isolation, maintenance, and scaffold cell-seeding

Peripheral blood for polymorphonuclear neutrophils’ (PMN) isolation was collected from healthy donors (*n* = 3) following informed consent. PMN were isolated from heparinized (10 U/mL) peripheral blood using Percoll density gradients (GE Healthcare, Chicago, USA) as previously described [35]. Briefly, two discontinuous gradients, 1.079 and 1.098, were used for PMN isolation with concomitant erythrocyte lysis (0.83% ammonium chloride containing 1% potassium bicarbonate, 0.04% ethylenediaminetetraacetic acid, and 0.25% bovine serum albumin). Isolated cells were resuspended in PBS and a viability of > 98% was determined. PMNs were seeded (1 × 10^5^/mL) on functionalized or unfunctionalized PLCA scaffolds and incubated for 30 min at 37 °C with 5% CO_2_.

Peripheral blood mononuclear cells (PBMC) were isolated from heparinized (10 U/mL) blood by centrifugation on Ficoll-Paque^TM^ Plus (GE Healthcare) as previously described [36]. PBMC were resuspended in Iscove’s modified Dulbecco’s medium (Sigma-Aldrich, St. Louis, USA) supplemented with 2.5% human AB serum (BioSera, France), 100 μg/mL streptomycin, 100 U/mL penicillin, 2mM l-glutamine (all from Sigma-Aldrich). PBMC were seeded (1 × 10^5^/mL) on functionalized or unfunctionalized PLCA scaffolds and incubated for 2 h at 37 °C with 5% CO_2_ to obtain adherent monocytes. After 2 h, the medium containing non-adherent cells was replaced with a fresh medium. The adherent monocytes were then incubated for 5 days at 37 °C with 5% CO_2_ to allow differentiation into macrophages.

#### *Escherichia coli* lipopolysaccharide (*E. coli* LPS) stimulation

After 30 min of incubation, PMN cultured on functionalized or unfunctionalized were treated with *E. coli* serotype O26:B6 LPS (Sigma-Aldrich L5543; Sigma-Aldrich) at 100 ng/mL. PMN were cultured in the presence of *E. coli* LPS for 4 h at 37 °C with 5% CO_2_ prior to downstream analysis. After 5 days of incubation, adherent macrophages from PBMC cultured on functionalized or unfunctionalized scaffolds were treated with *E. coli* serotype O26:B6 LPS (Sigma-Aldrich) at 100 ng/mL and incubated for 6 h at 37 °C with 5% CO_2_ prior to downstream analysis.

### In vitro ELISA

Total protein was isolated from in vitro scaffolds seeded with PMN. Briefly, culture media was removed from scaffolds and PMN were washed twice with cold PBS. Scaffolds with PMN were incubated at 4 °C for 20 min with RIPA buffer (Thermo Scientific). After sonification for 1 min and centrifugation at 16,000*g* at 4 °C for 20 min, the supernatant was collected and stored at 80 °C until use. The galectin-3 levels were quantified in collected supernatant using human galectin-3 immunoassay (Human Galectin-3 Quantikine ELISA Kit DGAL30, R&D Systems, Minneapolis, USA), according to the manufacturer’s instructions.

### In vivo inflammatory response and bone regeneration evaluation

Results from in vitro analyses lead to the preselection of PLCA scaffolds functionalized with RG-I dearabinated (PA scaffolds) for further in vivo experiments.

#### Rat subcutaneous model

Two incisions (~ 2 cm) were made on the back of 6–8-week-old Wistar rats after being anesthetized using isoflurane (Isoba® vet) (Schering Plough, NJ, USA). A pouch was dissected on each side and the unfunctionalized PLCA scaffolds (Control) or functionalized PLCA scaffolds (PA) were implanted subcutaneously and randomly distributed among all rats (at least *n* = 5 rats per time point). Wounds were sutured with Vicryl 4-0, and the animals were given buprenorphine (Temgesic® 0.3 mg/kg) subcutaneously as analgesic. Animals were euthanized with CO_2_ overdose at 4 days (acute inflammatory response) and 4 weeks (chronic inflammatory response) after implantation. The samples were harvested and stored in RNAlater (Invitrogen, Carlsbad, CA, USA) at − 80 °C until processed.

#### Calvaria bone defect model in aged rats

Aged Wistar rats (11–12 months old) were used to evaluate the inflammatory response and bone formation promoted by the functionalized scaffolds in an environment comparable to aged patients. Rats were anesthetized with isoflurane (Isoba® vet). Using aseptic techniques, a 2-cm anteroposterior cranial skin incision was made along the midline. The subcutaneous tissues and periosteum were dissected before a full-thickness defect (5-mm diameter) was created in the central area of each parietal bone using a trephine drill. Unfunctionalized PLCA scaffolds (Control), functionalized PLCA scaffold (PA), or autograft (Auto) were implanted in the defects before the periosteum and skin were repositioned and sutured with Vicryl 4-0. The autograft was cut into four fragments before being placed in the contralateral defect of the same animal. The autograft group aims to represent the autologous bone grafting technique. The animals were given buprenorphine (Temgesic® 0.3 mg/kg) subcutaneously as analgesic. After 2 weeks, animals were euthanized with CO_2_ overdose.

### In vitro and in vivo gene expressions using real-time RT-PCR

Total RNA was isolated from in vitro scaffolds seeded with PMN or macrophages using Trizol (Sigma) and the Qiagen RNeasy Mini Kit according to the manufacturer’s instructions. Using a tissue RNA isolation kit (Maxwell®, Promega, Madison, USA), total RNA was isolated from in vivo scaffolds. Quantity and purity were checked using a Nanodrop spectrophotometer (Thermo Fisher Scientific). RNA (300 ng) was reverse transcribed using a high-capacity complementary DNA reverse transcription kit (Applied Biosystems, CA, USA). Quantitative real-time PCR was conducted on a Light Cycler 480 SYBR Green I Master real-time PCR instrument (Roche Diagnostics GmbH). Target genes for in vitro and in vivo experiments were tumour necrosis factor-alpha (TNF-α), interleukin-1 beta (IL-1ß), interleukin-1alpha (IL-1α), interleukin-8 (IL-8), interleukin-10 (IL-10), galectin-1 (Gal-1), galectin-3 (Gal-3) Toll-like receptor 2 (TLR2), Toll-like receptor 4 (TLR4), interleukin-6 (IL-6), transforming growth factor beta-1 (TGF-ß) and colony-stimulating factor-1 receptor (CSF1R). The comparative 2^−ΔΔCt^ method was performed for analysis of relative gene expression data, as previously described [37]. Relative expressions were calculated after normalization to housekeeping genes, beta-2-microglobulin (B2M) for in vitro and glyceraldehyde 3-phosphate dehydrogenase (GAPDH) for in vivo.

### In vivo cytokine analysis using multiplex fluorescent bead-based immunoassay

Protein was isolated from harvested Control and PA scaffolds from the in vivo subcutaneous model by incubating them under shaking conditions at 4 °C for 20 min with RIPA buffer (Thermo Scientific), 1 × Halt™ protease inhibitor cocktail, and 1 × Halt™ phosphatase inhibitor cocktail (Thermo Scientific). After sonication for 5 min and centrifugation at 16,000*g* at 4 °C for 20 min, collected supernatant was quantified for protein using BCA assay (Pierce® BCA Protein assay kit, Thermo Scientific, Rockford, USA), following the manufacturer’s instructions. Standardized protein amounts were used in a Bio-Plex Rat 23-plex kit (Catalogue #12005641) (Bio-Rad, CA, USA) using the Luminex platform (Luminex®) for the processing of the Bio-Plex® 200 systems according to the manufacturer’s instructions. The amount of protein in each sample was extrapolated and compared with the standard curve ranges with concentrations reported in pg/mL.

### Histology and descriptive semi-quantitative histological evaluation

Retrieved samples from the in vivo subcutaneous model were fixed in 4% paraformaldehyde, before decalcification using 10% EDTA (Merck & Co, White House Station, NJ, USA) and paraffin embedding. Sections of 3–4 μm were stained with hematoxylin/eosin (Sigma, St. Louis, MO, USA). Qualitative and semiquantitative histological evaluation was carried out to assess the tissues’ response to the implanted scaffolds. Sections were evaluated blindly by two researchers independently under a light microscope (Leica, Solms, Germany). The infiltration of inflammatory cells inside the scaffold and that in direct contact with it, as well as the presence and quality of fibrous capsules were randomly evaluated in six fields of vision of each section (magnification 400×) using a scoring system we previously reported [32]. Infiltrated cells evaluated were those involved in acute inflammatory responses (neutrophils and plasma cells) and those involved in chronic responses (lymphocytes and foreign body giant cells).

### Statistical analyses

Data are presented as the mean values ± standard error of the mean (SEM). Significant differences were tested using one-way ANOVA, followed by a multiple comparison Tukey test using SPSS version 22 (IBM, NY, USA). A p value ≤ 0.05 was considered significant.

## Results

### PLCA scaffolds were successfully functionalized with plant derived nanoparticle RG-I in unmodified (PU) and modified (PA) form

#### Atomic force microscopy

**Fig. 1:**
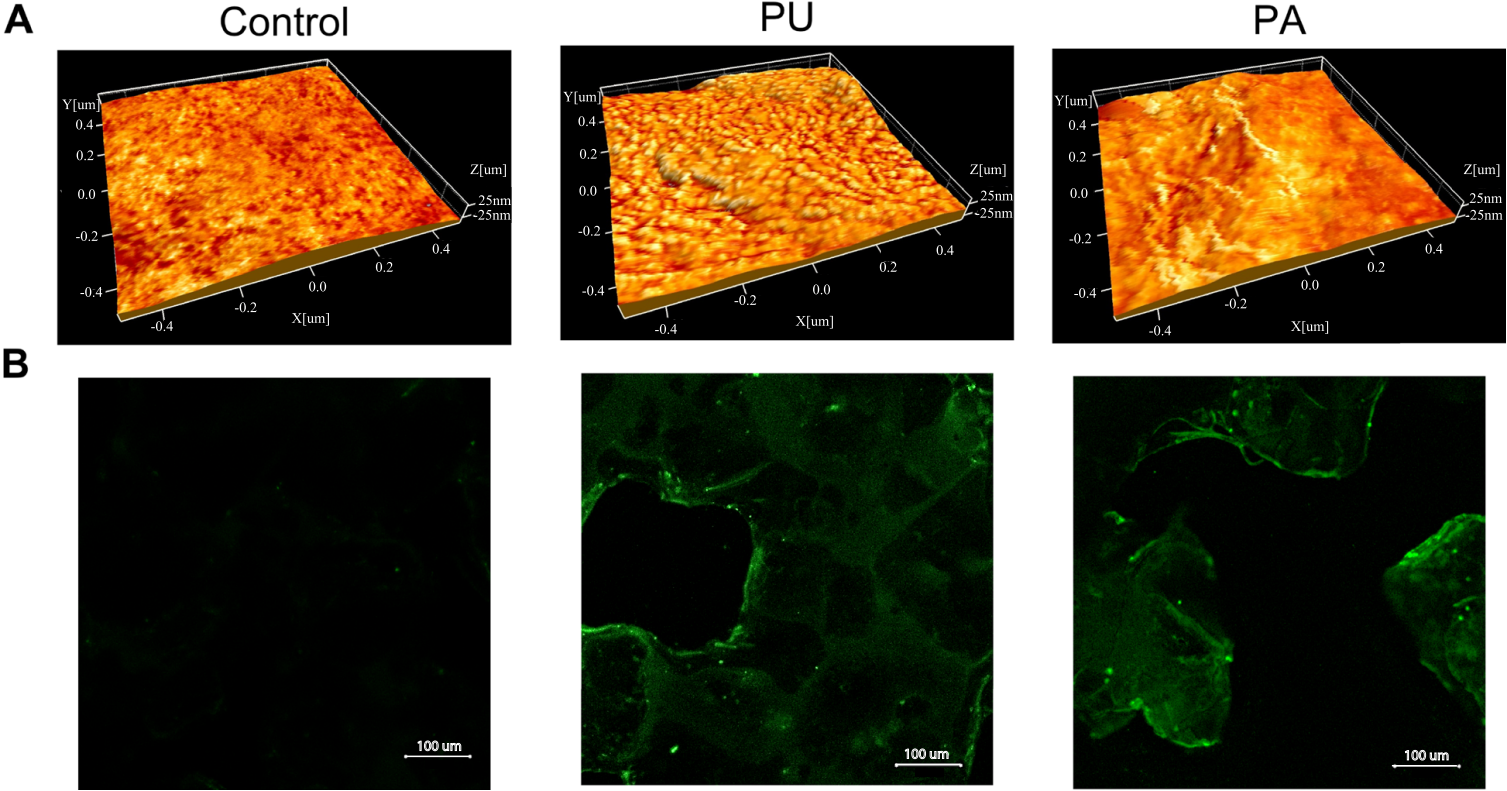
(**A**) Representative 3D images of unfunctionalized PLCA (Control), PU functionalized PLCA (PU), PA functionalized PLCA (PA) measured with intermittent imaging technique, multimode atomic force microscopy (AFM) 1μm Å ~ 1 μm with height range (topographical Peak-Valley) of approximately 50 nm and 512 × 512 pixels, corresponding to a theoretical lateral resolution of approximately 2 nm. PU, potato unmodified; PA, potato dearabinated; PLCA, poly(l-lactide-co-ɛ-caprolactone). (**B**) Representative confocal images of unfunctionalized PLCA scaffold (Control), functionalized PLCA scaffold with PU (PU), and functionalized with PA (PA)

Particles collected from the various PLCA scaffolds were between 200 and 600 μm in size. The functionalized and unfunctionalized scaffolds were analysed on an area of 1 μm × 1 μm each with a typical height range (topographical Peak-Valley) of approximately 50 nm and 512 × 512 pixels, corresponding to a theoretical lateral resolution of approximately 2 nm. The colour map of the 3D images is scaled to the phase contrast of the intermittent scan mode and superimposed on the 3D topographic data. The 3D analyses allowed to measure the overall height variation that was approximately ± 25 nm, without showing outstanding features such as grooves or protruding lines (Fig. 1A).

The unfunctionalized PLCA scaffold (Control) showed a small range of contrast variations with individual speckles and slightly brighter or lower shades of the orange background. In the PU functionalized PLCA scaffold (PU), the same background variation can be seen as in the control, however with sharper features of higher contrast distributed over the area. These features are approximately between 10–20 nm long and 5–10 nm wide. The PA functionalized PLCA scaffold (PA) showed a linear pattern of coating, and a line in the centre of the image approximately 450 nm long and 5–10 nm wide is clearly visible.

#### Confocal microscopy

The confocal images showed the presence of PU and PA on the functionalized PLCA scaffolds surface compared to Control (Fig. 1B). The regions of the scaffold coated with PU showed less intense fluorescence compared to the PA-coated scaffold. The higher fluorescence emission indicates the presence of a higher amount of galactan domains on the scaffold coated with PA.

### PLCA scaffolds functionalized with PU and PA modulated the inflammatory response of PMN and macrophages in vitro

#### Response of early and late inflammatory cells under *E. coli* LPS stimulation

**Fig. 2:**
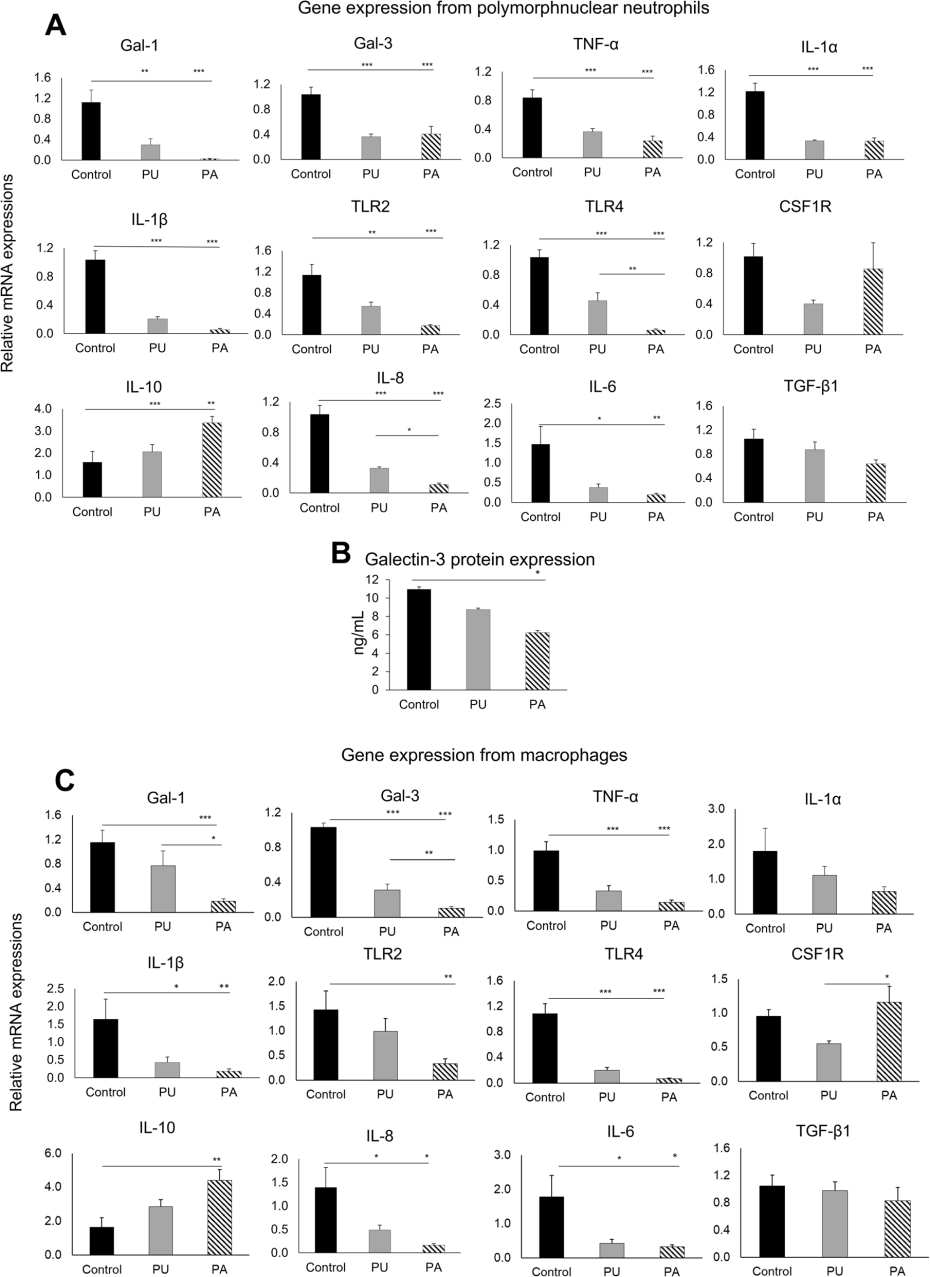
(**A**) Relative mRNA expression of selected genes expressed in the LPS-stimulated human polymorphonuclear neutrophils (PMN) cultured on Control (unfunctionalized) scaffolds, scaffolds coated with unmodified RG-I (PU) or on scaffolds coated with dearabinated RG-I (PA). (**B**) Galectin-3 levels quantified in LPS- stimulated PMN cultured on unfunctionalized (control) PLCA scaffolds, PU or PA-functionalized scaffolds. (**C**) Relative mRNA expression of selected genes expressed in the LPS-stimulated human monocyte-derived macrophages cultured on unfunctionalized PLCA scaffolds (Control), functionalized PLCA scaffolds with PU, and with PA. Data presented as fold change normalized to B2M (*p < 0.05, **p < 0.01, ***p < 0.001)

The real-time PCR data revealed the downregulation in PU and PA scaffolds of the pro-inflammatory genes tested and the upregulation of the anti-inflammatory gene IL-10 (Fig 2). Relative to the control scaffolds (Control), the differences in expression among all tested genes were highly significant except for TGF-β1 and CSF1R from PMN and IL-1α and TGF-β1 expression from macrophages.

The expression levels of all selected genes in the LPS-stimulated PMNs cultured on scaffolds functionalized with PU and PA were comparable. However, the expression of TLR4 and IL-8 was significantly lower in cells from the scaffolds coated with PA compared to PU. Galectin 3 (Gal-3) was downregulated in PU and PA compared to Control, with lower expression in PU compared to PA albeit insignificant. LPS-stimulated PMN showed a significantly decreased intracellular accumulation of galectin-3 in the presence of PA functionalization compared to Control (*p* < 0.001) (Fig. 2B). However, scaffold functionalized with PU did not significantly affect the level of galectin-3 produced by LPS-stimulated PMN (9 ng/mL) when compared with Control scaffolds (11 ng/mL).

In monocyte-derived macrophages the expression of most of the evaluated inflammatory genes was reduced on PU and PA functionalized scaffolds compared to Control (Fig. 2C). The expressions of Gal-1 and Gal-3 were significantly lower in the PA compared to the PU group. By contrast, gene expression of the anti-inflammatory markers IL10 and CSF1R showed a reversed pattern, where PA significantly upregulated the expression of these genes compared to the Control.

### PLCA scaffolds functionalized with PA modulated the host response in vivo towards an anti-inflammatory response at early and late time points

Our results indicated a positive effect of PA functionalization compared to PU on neutrophils and macrophages cultured in vitro. Therefore, the PA scaffold group was pre-selected to be further evaluated in the in vivo studies.

#### Gene expression of early and late inflammatory responses after 4 days and 4 weeks in vivo

**Fig. 3:**
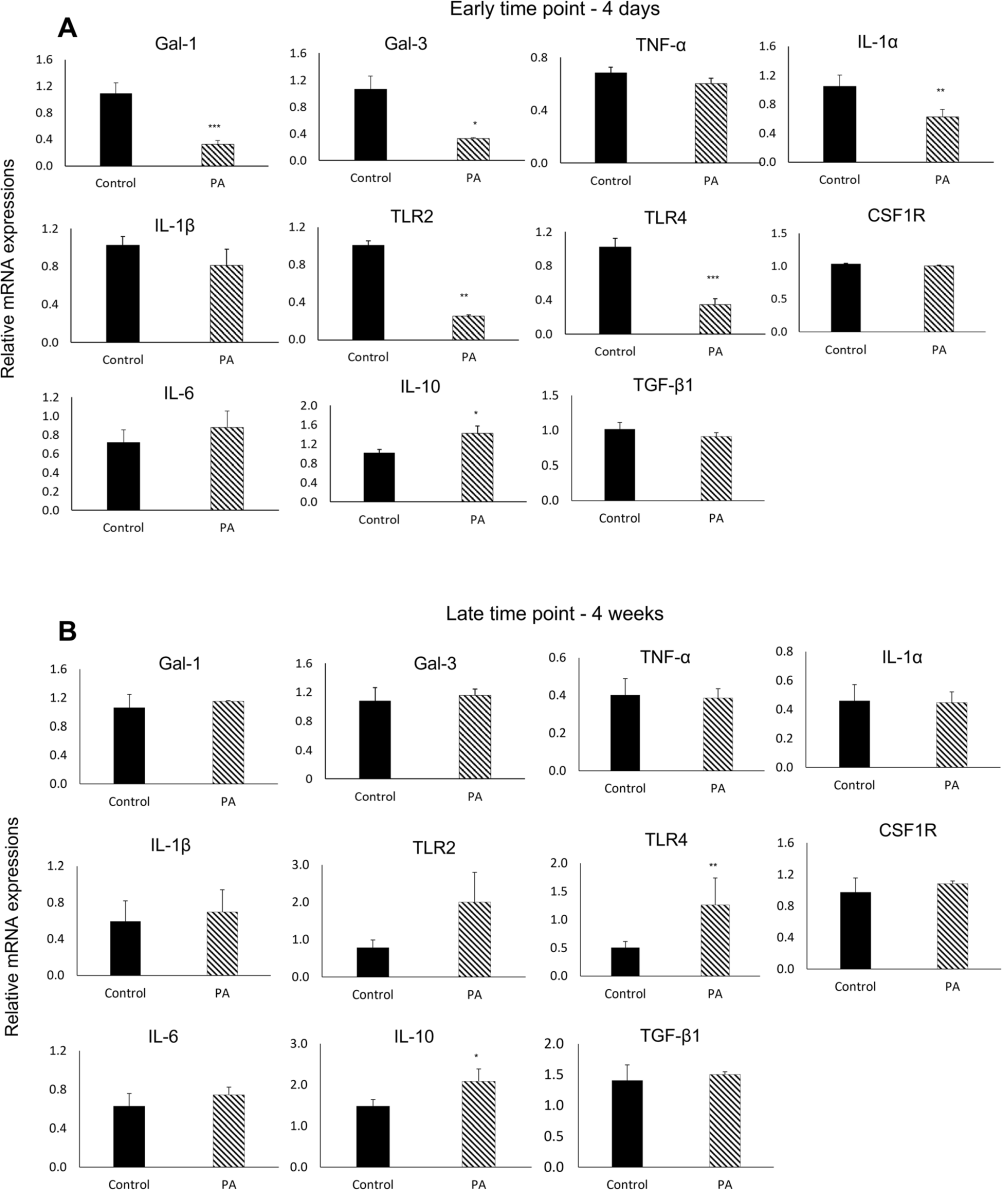
(**A**) Relative mRNA expression after 4 days in vivo of selected genes from unfunctionalized PLCA scaffolds (Control) and PA functionalized scaffolds. (**B**) after 4 weeks in vivo. Data are presented as fold change normalized to GAPDH. (*p < 0.05, **p < 0.01, ***p < 0.001).

In vivo gene expression showed comparable trends to the in vitro gene expressions (Fig. 3 A and B). In the acute inflammatory phase, PA functionalized scaffolds exhibited a general downregulation of pro-inflammatory genes, including Gal-1, Gal-3, TNF-α, IL-1α, IL-1β, TLR2, and TLR4 (Fig. 3A). Relative to the Control group, the differences among pro-inflammatory genes were significant only for Gal-1, Gal-3, TLR2, and IL-1α.

On the other hand, and in contrast to the in vitro results, PA slightly upregulated the expression of pro-inflammatory marker IL-6, albeit insignificant. The expressions of CSF1R and TGF-β1 from PA and Control were comparable. In contrast to the pro-inflammatory markers’ expressions, PA significantly increased the expression of the anti-inflammatory marker IL-10. Therefore, PLCA scaffolds functionalized with PA reduced the expression of pro-inflammatory markers and significantly promoted the anti-inflammatory markers’ expression in acute inflammatory response compared to Control.

In the late time point, after 4 weeks, the level of gene expression was comparable between Control and PA (Fig. 3B). In contrast to the trend seen in both in vitro and day 4 in vivo, PA functionalized scaffolds exhibited slight upregulation of pro-inflammatory TLR2 and TLR4 (*p* < 0.01) at week 4. After 4 weeks in vivo, the level of the anti-inflammatory marker IL-10 was significantly upregulated in PA. The mRNA expression of IL-10 was highly upregulated in PA during the acute inflammatory response and remained highly expressed in the late response as well.

#### A reduced foreign body reaction and fibrous capsule formation was observed when PLCA scaffolds functionalized with PA were implanted in vivo subcutaneously.

**Fig. 4:**
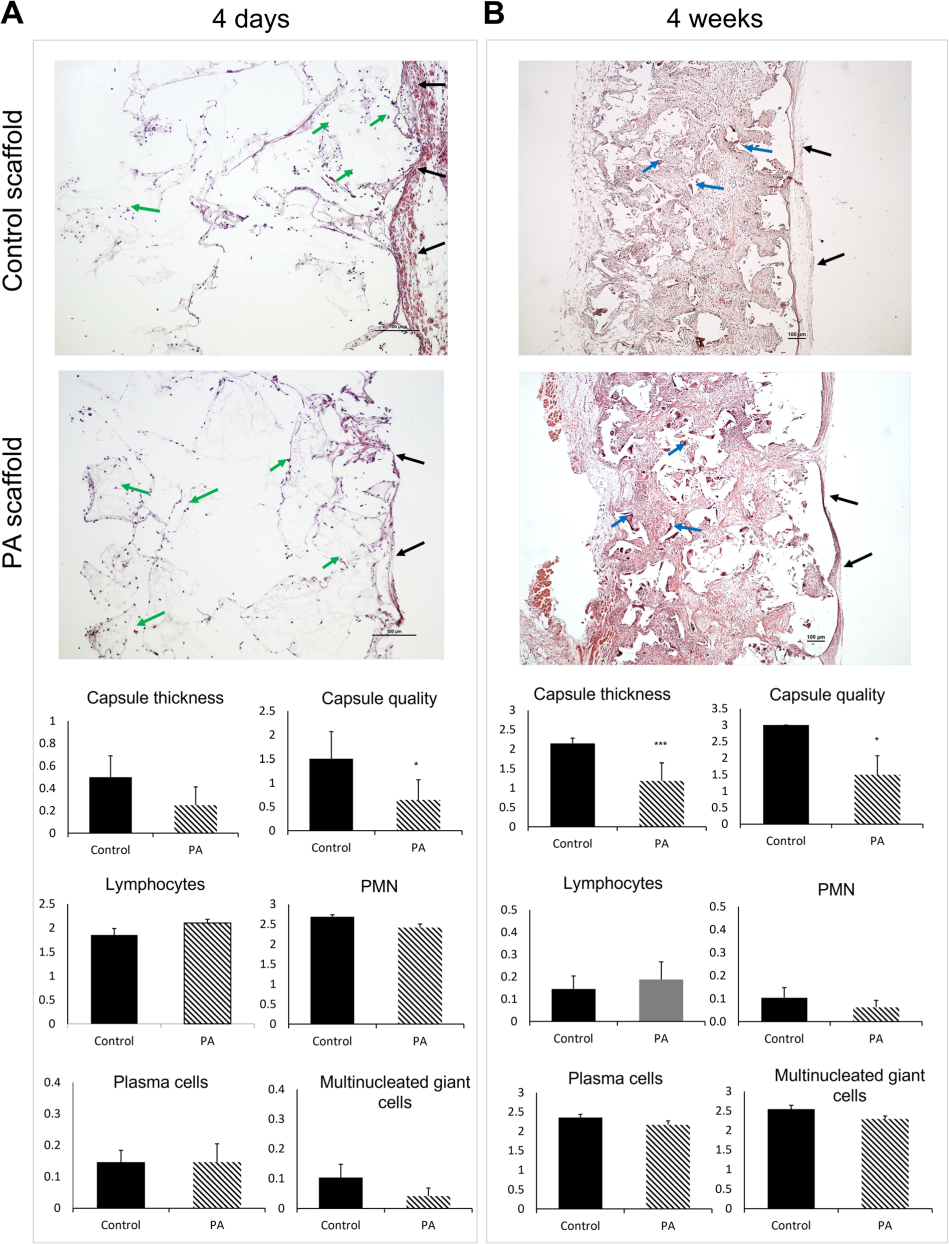
(**A**) Upper panel: Representative micrographs of hematoxylin/eosin-stained sections from unfunctionalized PLCA scaffolds (Control) and PLCA scaffolds functionalized with PA (PA) at 4 days. Magnification 100×. Lower panel: Histological grading of fibrous tissue capsule and inflammatory cell infiltration at day 4 (early inflammatory response) post implantation of Control and PA scaffolds. Data expressed as average score (*p < 0.05). (**B**) Upper panel: Representative micrographs of hematoxylin/eosin-stained sections from Control scaffolds and PA scaffolds at 4 weeks. Magnification 400×. Lower panel: Histological grading of fibrous tissue capsule and inflammatory cell infiltration at 4 weeks post-implantation (late inflammatory response) Control and PA scaffolds. Data expressed as average score (*p < 0.05)

The inflammatory response after scaffold implantation was also examined histologically after 4 days and 4 weeks to evaluate early and late acute host response respectively. In the acute host response, the fibrous encapsulation was more prominent in the control PLCA scaffold group (Control) compared to PA (Fig. 4A, black arrows). Most of the Control scaffolds were surrounded by fibrous capsules with a maximum thickness of 4 layers. No inflammatory cells were microscopically detected in the capsule formed around Control scaffolds. However, few inflammatory cells, mainly represented by lymphocytes were detected in the capsule surrounding PA scaffolds at 4 days. The quantity and diversity of inflammatory cells infiltrated into the scaffold pores were comparable between Control and PA groups (no significant differences), and the lymphocytes and PMNs were the main inflammatory cells recruited in the acute phase both at the periphery and the centre of the scaffold (Fig. 4A, green arrows). Very few multinucleated foreign body giant cells were observed at day 4 in both scaffold groups in comparison to the late time point.

At week 4 a fibrous encapsulation was seen in both Control and PA groups, however, compared to the early phase, Control and PA functionalized scaffolds developed a capsule composed mainly of fibroblasts. Consistent with the early time point, the PA scaffold group showed a significantly reduced thickness of the capsule than the Control group after 4 weeks **(**Fig. 4B, black arrows). The scaffold pores from both groups were infiltrated with fibrous connective tissue made of mature fibroblasts and blood vessels (Fig. 4B). Moreover, the lymphocytes and PMNs were replaced with foreign body giant cells (FBGC) and plasma cells, with more FBGC observed close to the scaffold structure in both Control and PA scaffolds (Fig. 4B). The PA group was observed to recruit a comparable number of inflammatory cells to the Control, with no significant differences.

#### PLCA scaffolds functionalized with PA modulated the cytokine and chemokine expressions in vivo during early and late inflammatory phases

**Fig. 5:**
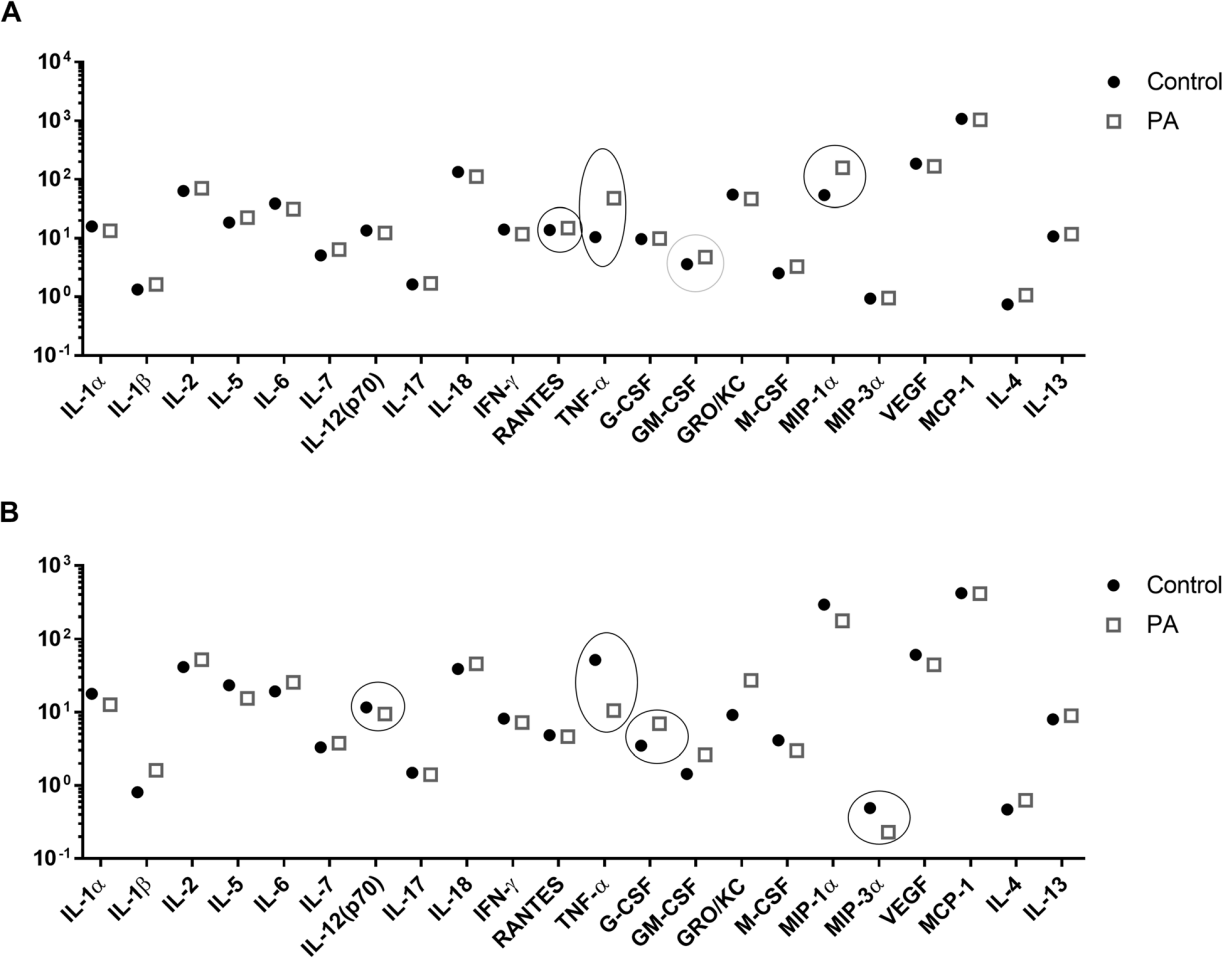
Cytokine profile from subcutaneously implanted unfunctionalized (Control) and functionalized (PA) scaffolds (**A**) 4 days and (**B**) 4 weeks post-implantation. Data are presented on a logarithmic scale (Log 10). Statistically significant readings are highlighted by circles. Black circle *p* < 0.05, Grey circle *p* < 0.01

The expression of all evaluated proteins after 4 weeks was remarkably lower compared to the expressions in the early phase response, after 4 days (Table S1 and S2). However, TNF-α increased by four folds on Control scaffolds at 4 weeks compared to 4 days, while on PA the expression was opposite (Fig. 5 A and B). TNF-α after 4 days was significantly lower on Control scaffolds compared to PA (*p* = 0.05), while at 4 weeks, it was significantly higher (5-fold) on Control compared to PA (*p* = 0.028). The expression of macrophage inflammatory protein 1-alpha MIP-1α from Control scaffolds was increased (6-fold) after 4 weeks compared to 4 days, while from PA scaffolds, the levels remained the same. Within the 4 days’ time point, MIP-1α was significantly higher on the PA scaffold compared to Control (*p* = 0.02), while within the 4 weeks’ time point, it was expressed twice as high from the Control scaffold compared to PA scaffold (Fig. 5 A and B) (Table S1 and S2).

After 4 days, the chemokine regulated on activation, normal T cell expressed and secreted (RANTES) (*p* = 0.043) and GM-CSF (*p* = 0.009) were significantly higher on PA scaffolds compared to Control. After 4 weeks only G-CSF was significantly higher on PA compared to Control (*p* = 0.03), while MIP-3α (*p* = 0.036) and IL-12 (*p* = 0.016) were found to be significantly lower on PA scaffolds compared to Control (Fig. 5 A and B) (Table S1 and S2).

### PLCA scaffolds functionalized with PA implanted in calvaria defects of aged rats downregulating pro-inflammatory gene markers while promoting osteogenic markers after 2 weeks in vivo

**Fig. 6:**
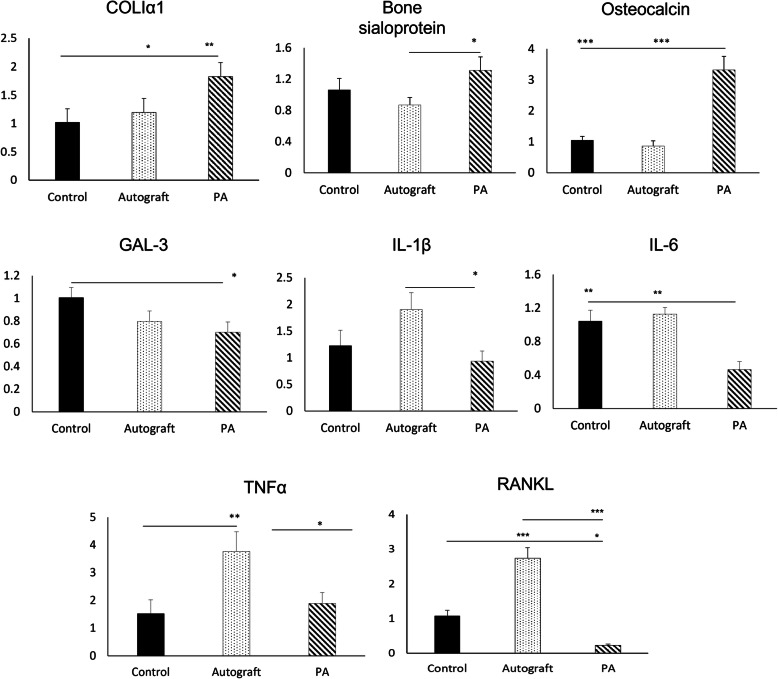
Relative mRNA expression of selected inflammatory and early bone-forming markers from different groups implanted in aged rat calvaria bone defects after 2 weeks. Unfunctionalized scaffold (Control), functionalized scaffold (PA), and autograft. Data are presented as fold change normalized to GAPDH (*p < 0.05, **p < 0.01, ***p < 0.001)

The in vivo gene expressions from calvaria bone defects in aged rats showed upregulation of osteogenic genes and downregulation of pro-inflammatory genes on PA scaffolds compared to autograft and/or Control scaffolds (Fig. 6). The osteogenic markers, collagen, type I, alpha 1 (COL-Iα1) and osteocalcin, were significantly upregulated on PA functionalized scaffolds compared to autograft and Control scaffolds, while bone sialoprotein was significantly upregulated on PA functionalized scaffold compared to autograft only. The receptor activator of nuclear factor kappa-B ligand (RANKL) was significantly downregulated and minimally expressed on PA functionalized scaffolds compared to Control and autograft. Furthermore, the autograft group stimulated high gene expressions of RANKL, but also the inflammatory markers, TNFα, IL-6, and IL-1β. The inflammatory marker, IL-1β was significantly downregulated on PA functionalized scaffold compared to autograft and IL-6 was significantly downregulated on PA compared to autograft and Control scaffolds. The TNFα was significantly downregulated on PA functionalized scaffold and Control compared to autograft.

## Discussion

In this study, we successfully functionalized PLCA scaffolds with RG-I and confirmed its ability to modulate inflammation both in vitro and in vivo, while promoting bone regeneration in an aged rodent model.

PLCA scaffolds functionalized with RG-I modulated pro- and anti-inflammatory markers in vitro and in vivo at the gene level, which were translated to protein and expressed in cytokine levels detected in vivo. Furthermore, our results suggested that plant-derived RG-I with a relatively higher amount of galactose (PA) than arabinose may play a crucial role in the modulation of inflammatory response induced by bacterial LPS. The inhibition of pro-inflammatory and stimulation of anti-inflammatory markers by PA functionalized PLCA scaffolds resulted in an increased level of osteogenic genes in the calvaria rat model compared to control. In addition, the RANKL and TNF-α expression on PA functionalized PLCA scaffolds was significantly lower compared to Autograft, indicating a decreased risk of resorption and chronic inflammation.

In our in vitro experiments, the pro-inflammatory response from PMN and macrophages was significantly reduced on PA functionalized PLCA scaffold compared to control, by downregulation of mRNA expressions of inflammatory markers, such as IL6, TNF-α, IL8, IL1α, and IL1β. Interestingly, the anti-inflammatory response was significantly induced by upregulation of IL-10 expression in the PMN and macrophages cultured. These results suggest that RG-I may modulate early (24–48 h) and late (2–7 days) inflammatory processes in vitro. Furthermore, the findings showed that modified RG-I with a relative higher content of galactose (PA) had a stronger effect on gene expression compared to unmodified RG-I (PU). The in vivo results after 4 days and 4 weeks confirmed anti-inflammatory properties of PA functionalized PLCA scaffolds as the IL-10 expression was significantly increased compared to control. Several studies demonstrated that cell response is affected by the chemical and structural composition of RG-I side chains [13–15, 17, 18, 38]. Pectins have also shown to exert immune effects via interaction with pattern recognition receptors, such as Toll-like receptors [39]. In fact, a previous study postulated that the highly branched side chains of pectins are essential for their anti-inflammatory properties on IL-6 secretion from LPS-induced macrophages [40]. The structural part of RG-I that has been identified to modulate immune responses is the galactan side chain, which binds to the carbohydrate recognition domain of galectins [41]. Galectin-3 (Gal-3) is particularly expressed on immune cells, such as monocytes, macrophages, neutrophils, and epithelial cells [42]. These immune cells use Gal-3 as a pattern recognition receptor to induce innate immune responses against pathogens, such as bacterial infection [43]. The galactan side chain of RG-I may enhance the anti-pathogenic effect by binding to Gal-3 [39, 44]. In fact, our results of Gal-3 expression at protein level from PMN culture showed a significant decrease in its accumulation intracellularly in the presence of PA. Furthermore, mRNA expression of Gal-3 and Gal-1 was significantly reduced in macrophage cultures on PA compared to PU scaffolds. However, when PLCA scaffolds were previously functionalized with demineralized dentin matrix, the levels of IL-6 and IL-8 mRNA from bone marrow–derived stem cells were increased compared to control [33]. This confirms the role played by RG-I and galectins in damping the inflammatory response from immune cells.

In addition, downregulation of pattern recognition receptors, TLR-2 and TLR-4 was observed in both neutrophils and macrophages cultured on the PA scaffold. This is in line with several studies that demonstrated that pectins inhibit LPS-induced TLR-2 and TLR-4 activation in monocytes, which has been proposed to be via its galactan binding mechanism [40, 45]. Our in vitro findings of downregulated Gal-3 and TLR-4 in monocytes, leading to upregulation of IL-10 gene expressions, confirms a direct interaction between Gal-3 and TLR-4. Similar findings were reported previously in an inflammatory mouse model suggesting that their inhibition promotes anti-inflammatory effects [46]. Furthermore, high levels of Gal-3 expression because of bacterial infection drives neutrophil infiltration and production of pro-inflammatory cytokines [47–50]. Therefore, it can be hypothesized that inhibition of Gal-3 by the galactan side chain may lead to a positive modulation of inflammation.

In our subcutaneous rat model, mRNA from harvested scaffolds showed significant downregulation of Gal-1, Gal-3, TLR-2, and TLR-4 expression after 4 days by PA-functionalized PLCA scaffolds. This is comparable to our in vitro results. However, this pattern of expression was not seen after 4 weeks in vivo. In fact, TLR-4 was significantly increased in PA scaffolds, compared to Control, which may suggest RG-I properties to only modulate acute inflammatory host responses. On the contrary, when PLCA scaffolds were previously functionalized with nanodiamond particles and their host response was evaluated subcutaneously in a mouse model, they showed an increased acute inflammatory response at gene level that was significantly downregulated after 8 weeks [32]. This difference in our in vitro and in vivo responses can also be explained by a difference in the experimental models since the animal model was not challenged by an LPS induction. Furthermore, modulation of acute responses observed with RG-I functionalization might be beneficial for the reduction of post-operative infection risks.

Furthermore, the cytokine level expressed in vivo from the harvested scaffolds, demonstrated the pro-inflammatory chemokine RANTES significantly lower on PA compared to Control after 4 days, suggesting a reduction in unwanted acute inflammatory responses. RANTES and MIP-1α, which have CCR5 and CCL3 as ligands respectively, are involved in pro-inflammatory responses and are targeted for therapeutics in chronic inflammatory diseases [51]. The RG-I structure was shown to mimic the glycosaminoglycans’ structure [52–54] and the presence of glycosaminoglycans has been reported to modulate the oligomerization of MIP-1α and RANTES via the N-termini of CC chemokines [55]. Therefore, PA could have affected the levels of MIP-1α and RANTES. Another significant differential expression at the protein level was seen from GM-CSF. Several pro- and anti-inflammatory mechanisms of action for GM-CSF were described and suggested that its action is determined by the presence or absence of other relevant cytokines [56]. In our results, GM-CSF’s concentration was expressed significantly higher on PA scaffolds compared to Control scaffolds, albeit its absolute concentration was relatively low compared to other cytokines. Therefore, the results need to be analysed in a holistic context relevant to the overall immune response.

The cytokines expressed from the different scaffolds after 4 weeks showed significantly higher levels of pro-inflammatory markers, IL-12 and TNF-α on Control scaffolds compared to PA scaffolds. In fact, both cytokines are secreted by activated macrophages and are involved in mechanisms of fibrosis [57]. These findings correlate with our histological observations, which indicated a thicker layer of fibrous capsule around the unfunctionalized Control scaffolds. Furthermore, MIP-3α was expressed significantly lower on PA scaffolds compared to Control scaffolds, which may support the reduction of chronic inflammation during late stages of bone formation [58].

Bone healing is highly dependent on the initial inflammatory phase after injury, which is affected by both local and systemic host responses to the surgical procedure and grafting biomaterial. The highly controlled pro-inflammatory and anti-inflammatory phases generated by the immune system are essential to create the conditions for successful bone tissue repair/regeneration. The healing response to a biomaterial is often initiated by fibrous encapsulation, where its progression is an indicator of the biocompatibility of the biomaterial [59]. The long-term host response to an implanted scaffold is affected by many factors, one of which is the scaffold degradation. The optimal degradation rate of the scaffold should correspond to the rate of bone tissue regeneration. Another important requirement is that the breakdown products of the degradation process must not be toxic without causing a prolonged foreign body reaction [60]. We found that PLCA scaffolds functionalized with PA significantly reduced the thickness of the fibrotic capsule formation in the rat subcutaneous model compared to Control scaffolds at week 4. Decreased capsule thicknesses facilitate cell infiltration and promote tissue regeneration. Numerous studies reported that the size of the fibrotic capsule can be regulated through the addition of different bioactive molecules such as growth factors and proteins to stimulate the regeneration process [59]. The observed slight decrease in the number of foreign body giant cells in PA functionalized PLCA scaffolds (at both time points) compared to Control may indicate a milder foreign body reaction. These histological observations in our current study have been coupled in our previous reports with a faster degradation for functionalized scaffolds with nanodiamonds compared to unfunctionalized PLCA scaffolds [32]. The degradation process of aliphatic polyesters such as PLCA occurs by bulk hydrolysis that causes cleavage of ester bonds and decrease in molecular weight. This is followed by a second phase characterized by foreign body giant cells that engulf the breakdown. Degradation analysis was not carried out for our functionalized PA scaffolds; however, we extensively studied the degradation of the unfunctionalized PLCA scaffolds previously both in vitro and in vivo [32, 61]. There, we reported that after almost 2–3 months, 60–70% of the number average molecular weight was decreased in vivo. This rate of degradation was accelerated when the PLCA scaffolds were functionalized to increase wettability [32]. Since RG-I is known to increase wettability of surfaces [14, 62], we postulate a faster hydrolysis of our functionalized PA scaffolds compared to Control. The degradation rate of polymeric scaffolds can be tailored by using different monomers in the copolymer or functionalizing with other hydrophilic factors [60, 61]. The scaffold’s porosity and pore size is also critical for degradation [60, 63]. Our PLCA scaffolds have interconnected porosity that was optimized previously [64, 65] for cell attachment, proliferation, and differentiation as well as new bone and capillary formation [66, 67].

Tuning and controlling degradation is not only important for the support corresponding to the rate of bone tissue regeneration, however it is also necessary for the release profile of RG-I. Controlling the release of bioactive factors can be carried out by either physically or chemically functionalizing the copolymer scaffolds [30]. In our study, we functionalized PLCA by simple physisorption, which is a functionalization method proven to burst an early release of the bioactive factor [30]. To control and instruct acute inflammation, an early release of the anti-inflammatory molecule might be beneficial. However, in other circumstances when bone-inducing molecules are to be released, a more sustained long-term release was shown to be more efficient [30]. Even though tuning the scaffold degradation and monitoring the release profile of RG-I was not in the scope of this study, it is warranted to be investigated in the future. This is because the release profile of RG-I can certainly influence the cellular activity and aids in regulating immunological responses further.

While the scientific evidence about anti-inflammatory properties of different origins of RG-I is limited and not uniform, the osteogenic properties have been described in several studies [13–15, 17, 18, 38]. Furthermore, most of these studies found that RG-I with a relatively higher amount of galactose stimulates osteoblasts to produce an extra-cellular matrix, followed by its mineralization and leading to bone formation. Bone regeneration is challenged in compromised patients, especially aged patients. Therefore, it is essential to know how ageing, alters inflammatory responses and regenerative processes. In this study, we further investigated the ability of RG-I to stimulate bone regeneration in a critical size bone defect in aged rats. Our results clearly showed superior osteogenic properties (upregulation of mRNA COL-1 and osteocalcin) in PA functionalized PLCA scaffolds compared to Control unfunctionalized scaffolds and autograft. Furthermore, the autograft group in our study stimulated high expressions of pro-inflammatory markers, IL-6, TNF-α, and IL-1α, which may amount to unpredicted resorption [8]. Indeed, our results revealed a 10-fold higher expression of RANKL on autograft, compared to PA functionalized scaffolds. These results are in line with clinical findings, showing a higher resorption rate of autogenous bone grafts compared to some slow degrading biomaterials [68]. Furthermore, the main challenge of autogenous bone grafting is limited availability and donor site morbidity [5]. In addition, elderly patients undergoing complex surgical procedures are at a higher risk of hospitalization due to complications related to bacterial infection [2, 3]. Therefore, there is a high demand for the development of an immunomodulating scaffold with osteoinductive and osteoconductive properties that will reduce the operating time and risk of complications.

The PLCA scaffold functionalized with PA has shown in this study to reduce the inflammatory state stimulated by bacterial LPS, which may contribute to the controlling of post-operative infection risks. Additional studies on multispecies biofilm conditions to assess further the immunomodulatory properties of these functionalized scaffolds are required, as well as longer time points on calvaria defects to investigate the quality of bone formed.

## Conclusion

We have shown that PLCA scaffolds functionalized with plant-derived RG-I with relatively higher amount of galactose play a role in the modulation of inflammatory responses both in vitro and in vivo subcutaneously and promote the initiation of bone formation in a critical-sized bone defect of an aged rodent. Taken together, our study has addressed the increasing demand in bone tissue engineering for immunomodulatory 3D scaffolds that promote osteogenesis and modulate immune responses by utilizing bioactive factors.

## Supplementary Information

**Additional file 1.**

## Data Availability

The datasets used and/or analysed during the current study are available from the corresponding author on reasonable request.

## Abbreviations

3D: Three-dimensional
RG-I: Rhamnogalacturonan-I
Gal: Galactose
PLCA: Poly(l-lactide-co-ɛ-caprolactone)
RG-I: Rhamnogalacturonan-I
PA: Potato RG-I dearabinated
PU: Unmodified potato RG-I
AFM: Atomic force microscopy
FITC: Fluorescein isothiocyanate
PMN: Polymorphonuclear neutrophils
PBMC: Peripheral blood mononuclear cells
*E. coli* LPS: *Escherichia coli* lipopolysaccharide
Auto: Autograft
TNF-α: Tumour necrosis factor-alpha
IL-1ß: Interleukin-1 beta
IL-1α: Interleukin-1 alpha
IL-8: Interleukin-8
IL-10: Interleukin-10
Gal-1: Galectin-1
Gal-3: Galectin-3
TLR2: Toll-like receptor 2
TLR4: Toll-like receptor 4
IL-6: Interleukin-6
TGF-ß1: Transforming growth factor beta-1
CSF1R: Colony-stimulating factor-1 receptor
B2M: Beta-2-microglobulin
GAPDH: Glyceraldehyde 3-phosphate dehydrogenase
SEM: Standard error of the mean
MIP-1α: Macrophage inflammatory protein 1-alpha
RANTES: Chemokine regulated on activation, normal T cell expressed and secreted
COL-Iα1: Collagen, type I, alpha 1
RANKL: Receptor activator of nuclear factor kappa-B ligand
