# Supplementary material for: Immune-instructive copolymer scaffolds using plant-derived nanoparticles to promote bone regeneration
Source: Inflamm Regen. 2022 Apr 3;42:12. doi: 10.1186/s41232-022-00196-9 (PMC8977008; doi:10.1186/s41232-022-00196-9)
Supplement: Supplementary file 1 — Additional file 1. [file 41232_2022_196_MOESM1_ESM.docx]

Supporting Information

**Immune-instructive copolymer scaffolds using plant-derived nanoparticles to promote bone regeneration**

Salwa Suliman*, Anna Mieszkowska, Justyna Folkert, Neha Rana, Samih Mohamed-Ahmed, Tiziana Fuoco, Anna Finne-Wistrand, Kai ~~Dirchert~~ Dirscherl, Bodil Jørgensen, Kamal Mustafa, Katarzyna Gurzawska-Comis*

**Table S1.** Levels of the detected cytokines and chemokines after 4 days *in vivo*

| **Pro-inflammatory markers** | | | | | |
| --- | --- | --- | --- | --- | --- |
| **Marker** | **Group** | **Average Level (pg/ml)** | **SEM** | **P-value** |  |
| IL-1α | Control | 15.86 | 4.31 | 0.119 |  |
|  | PA | 13.36 | 2.44 |  |  |
| IL-1β | Control | 1.33 | 0.44 | 0.506 |  |
|  | PA | 1.62 | 0.48 |  |  |
| IL-2 | Control | 63.22 | 8.59 | 0.453 |  |
|  | PA | 70.69 | 12.27 |  |  |
| IL-5 | Control | 18.64 | 1.08 | 0.301 |  |
|  | PA | 22.45 | 1.13 |  |  |
| IL-6 | Control | 38.83 | 3.98 | 0.159 |  |
|  | PA | 31.11 | 6.65 |  |  |
| IL-7 | Control | 5.10 | 0.35 | 0.07 |  |
|  | PA | 6.43 | 1.29 |  |  |
| IL-12(p70) | Control | 13.49 | 2.77 | 0.346 |  |
|  | PA | 12.33 | 1.99 |  |  |
| IL-17 | Control | 1.63 | 0.24 | 0.862 |  |
|  | PA | 1.68 | 0.27 |  |  |
| IL-18 | Control | 133.36 | 45.53 | 0.760 |  |
|  | PA | 112.12 | 32.20 |  |  |
| IFN-ɣ | Control | 14.12 | 2.52 | 0.637 |  |
|  | PA | 11.67 | 3.02 |  |  |
| RANTES | Control | 13.76 | 2.14 | 0.043* |  |
|  | PA | 14.87 | 4.36 |  |  |
| TNF-α | Control | 10.40 | 5.47 | 0.05* |  |
|  | PA | 47.65 | 25.77 |  |  |
| **Cytokines/ Growth factors** | | | | | |
| **Marker** | **Group** | **Average Level (ng/ml)** | **SEM** | **P-value** |  |
| G-CSF | Control | 9.63 | 1.76 | 0.157 |  |
|  | PA | 9.89 | 2.80 |  |  |
| GM-CSF | Control | 3.59 | 0.49 | 0.009 ** |  |
|  | PA | 4.75 | 0.85 |  |  |
| GRO/KC | Control | 55.01 | 13.82 | 0.849 |  |
|  | PA | 46.01 | 12.50 |  |  |
| M-CSF | Control | 2.52 | 0.39 | 0.158 |  |
|  | PA | 3.26 | 0.69 |  |  |
| MIP-1α | Control | 53.69 | 7.79 | 0.02 * |  |
|  | PA | 158.43 | 46.25 |  |  |
| MIP-3α | Control | 0.93 | 0.20 | 0.950 |  |
|  | PA | 0.95 | 0.19 |  |  |
| VEGF | Control | 185.50 | 35.70 | 0.356 |  |
|  | PA | 167.20 | 46.72 |  |  |
| MCP-1 | Control | 1086.62 | 141.63 | 0.343 |  |
|  | PA | 1045.40 | 201.08 |  |  |
| **Anti-inflammatory markers** | | | | | |
| **Marker** | **Group** | **Average Level (ng/ ml)** | **SEM** | **P-value** |  |
| IL-4 | Control | 0.74 | 0.12 | 0.49 |  |
|  | PA | 1.06 | 0.32 |  |  |
| IL-13 | Control | 10.73 | 1.65 | 0.352 |  |
|  | PA | 11.66 | 2.08 |  |  |

*P<0.05, **P<0.01

**Table S2.** Levels of the detected cytokines and chemokines after 4 weeks *in vivo*

| **Pro inflammatory markers** | | | | | |
| --- | --- | --- | --- | --- | --- |
| **Marker** | **Group** | **Average Level (ng/ml)** | **SEM** | **P-value** |  |
| IL-1α | Control | 17.84 | 2.44 | 0.220 |  |
|  | PA | 12.59 | 4.26 |  |  |
| IL-1β | Control | 0.81 | 0.27 | 0.155 |  |
|  | PA | 1.62 | 0.54 |  |  |
| IL-2 | Control | 41.68 | 5.55 | 0.090 |  |
|  | PA | 52.19 | 22.02 |  |  |
| IL-5 | Control | 23.31 | 2.76 | 0.330 |  |
|  | PA | 15.44 | 3.49 |  |  |
| IL-6 | Control | 19.33 | 5.04 | 0.061 |  |
|  | PA | 25.68 | 1.02 |  |  |
| IL-7 | Control | 3.29 | 0.89 | 0.537 |  |
|  | PA | 3.77 | 1.21 |  |  |
| IL-12 (p70) | Control | 11.58 | 2.86 | 0.016 * |  |
|  | PA | 9.46 | 1.39 |  |  |
| IL-17 | Control | 1.50 | 0.16 | 0.555 |  |
|  | PA | 1.41 | 0.12 |  |  |
| IL-18 | Control | 38.94 | 11.92 | 0.648 |  |
|  | PA | 45.75 | 12.46 |  |  |
| IFN-ɣ | Control | 8.15 | 2.18 | 0.079 |  |
|  | PA | 7.24 | 2.77 |  |  |
| RANTES | Control | 4.86 | 0.51 | 0.565 |  |
|  | PA | 4.62 | 0.82 |  |  |
| TNF-𝛂 | Control | 51.89 | 33.30 | 0.028 * |  |
|  | PA | 10.55 | 6.56 |  |  |
| **Cytokines/ growth factors** | | | | | |
| **Marker** | **Group** | **Average Level (ng/ml)** | **SEM** | **P-value** |  |
| G-CSF | Control | 3.47 | 0.78 | 0.03 * |  |
|  | PA | 6.96 | 2.51 |  |  |
| GM-CSF | Control | 1.44 | 0.46 | 0.08 |  |
|  | PA | 2.63 | 1.36 |  |  |
| GRO/KC | Control | 9.19 | 1.43 | 0.667 |  |
|  | PA | 27.24 | 18.49 |  |  |
| M-CSF | Control | 4.13 | 0.36 | 0.155 |  |
|  | PA | 2.99 | 0.80 |  |  |
| MIP-1α | Control | 294.25 | 37.20 | 0.421 |  |
|  | PA | 177.38 | 50.16 |  |  |
| MIP-3α | Control | 0.49 | 0.18 | 0.038 * |  |
|  | PA | 0.23 | 0.08 |  |  |
| VEGF | Control | 61.16 | 11.00 | 0.474 |  |
|  | PA | 44.61 | 18.78 |  |  |
| MCP-1 | Control | 421.24 | 22.17 | 0.103 |  |
|  | PA | 415.46 | 90.11 |  |  |
| **Anti-inflammatory markers** | | | | | |
| **Marker** | **Group** | **Average Level (ng/ml)** | **SEM** | **P-value** |  |
| IL-4 | Control | 0.47 | 0.05 | 0.32 |  |
|  | PA | 0.63 | 0.35 |  |  |
| IL-13 | Control | 7.99 | 1.61 | 0.810 |  |
|  | PA | 8.95 | 1.48 |  |  |

*P<0.05, **P<0.01
